# Beyond Social Media Use: A Cross-Sectional Study of Digital Engagement and Perceived eHealth Literacy Among Nursing Students

**DOI:** 10.3390/nursrep16070251

**Published:** 2026-07-18

**Authors:** Rana Alaseeri, Raghad Alshammari

**Affiliations:** Department of Basic Nursing Care, College of Nursing, Majmaah University, Al Majmaah 15341, Saudi Arabia

**Keywords:** social media use, eHealth literacy, nursing students, digital engagement, online health information, digital health

## Abstract

**Background/Objective:** Social media platforms have become increasingly integrated into nursing students’ academic and daily lives, influencing how health-related information is accessed, evaluated, and shared. At the same time, eHealth literacy has emerged as an important competency for nursing students within contemporary digital healthcare environments. This study aimed to examine the relationship between multidimensional social networking engagement and perceived eHealth literacy among undergraduate nursing students. **Methods:** A cross-sectional descriptive correlational design was used among undergraduate nursing students (N = 146). Data were collected using an electronic survey that included demographic characteristics, the Social Networking Usage Questionnaire, and the Revised eHealth Literacy Scale. Descriptive statistics, Pearson’s correlation, and linear regression analyses were performed using SPSS version 26. **Results:** Participants reported moderate-to-high social networking engagement and generally favorable perceived eHealth literacy. A strong positive correlation was identified between SNUQ and eHEALS-R scores (*r* = 0.75, *p* < 0.001), with academic use showing the strongest dimension-level association with perceived eHealth literacy. In the adjusted regression model, social networking engagement remained independently associated with perceived eHealth literacy, whereas demographic variables and daily social media use showed no significant associations. **Conclusions:** The findings suggest that how nursing students engage with digital platforms is associated with their confidence in accessing, evaluating, and applying online health information. Strengthening purposeful and critical digital engagement within nursing education may help prepare students for contemporary digital healthcare environments.

## 1. Introduction

Social media platforms have become increasingly integrated into nursing students’ academic and daily routines. Beyond social communication, these platforms are commonly used for academic discussions, collaborative learning, exam preparation, and rapid access to educational resources [1,2]. Nursing students also use social networking platforms to exchange experiences, discuss clinical topics, and seek educational support from peers [3]. As a result, digital engagement has become part of how many nursing students access, interpret, and interact with health-related information within contemporary learning environments.

Despite these potential educational benefits, the growing reliance on social media also raises several concerns within nursing education. The large volume of online information and the speed of digital communication may expose students to inaccurate or misleading health content, making it more difficult to evaluate the credibility and quality of online resources [4,5]. Previous studies have also linked excessive or unstructured digital use with academic stress, burnout, and problematic smartphone use among nursing students [6,7]. At the same time, more purposeful and academically focused use of social media has been associated with collaborative learning, peer support, and stronger academic engagement [1,3]. Existing evidence suggests that the educational influence of social media may depend more on how students engage with digital platforms than on the amount of time spent using them.

Within this evolving digital environment, nursing students are increasingly expected to develop strong eHealth literacy skills to safely navigate online health information and digital healthcare resources. eHealth literacy refers to the ability to seek, understand, evaluate, and apply electronic health information when making health-related decisions [8]. It also includes critical appraisal skills and the ability to distinguish reliable information from misinformation in online environments [5]. For nursing students, these competencies are particularly important because healthcare education and clinical practice increasingly rely on digital communication and evidence-based online resources [9]. As social media becomes more integrated into students’ educational and informational routines, greater attention has been directed toward understanding the factors associated with eHealth literacy among nursing students [1,3].

Recent studies have increasingly examined the relationship between social media use and digital health-related competencies among healthcare students. Some evidence suggests that social media may support learning experiences by facilitating communication, collaborative learning, and access to educational resources [3]. More organized and academically focused use of digital platforms has also been associated with greater engagement with educational content and improved access to health-related information [10]. Still, concerns remain regarding excessive or unstructured digital use. Previous studies have linked passive digital consumption and problematic smartphone use with negative psychological outcomes among nursing students [6], in addition to higher levels of academic stress and burnout [7]. Existing findings therefore continue to reflect both the educational opportunities and potential challenges associated with social media use among nursing students.

Nevertheless, evidence examining eHealth literacy among healthcare students continues to show considerable variation across findings. For instance, Ibrahim et al. [2] reported that demographic variables such as age and gender were not significantly associated with digital competencies or online health information use. In contrast, Panczyk et al. [11] reported differences in social media literacy across educational levels among nursing students, including skills related to online information evaluation [11]. Other studies have also suggested that individual academic and psychological characteristics may influence eHealth literacy levels within nursing education [8]. Overall, these variations across literature highlight the multidimensional nature of eHealth literacy within different educational and sociocultural contexts. 

Although international literature has increasingly explored social media use among nursing students, the relationship between multidimensional social networking engagement and perceived eHealth literacy remains underexamined. Previous studies have often treated social media use as a broad behavior or focused mainly on exposure time. More recently, a scoping review highlighted that social media engagement among nursing and midwifery students extends across educational, social, and professional contexts, with implications for professional identity and responsible online conduct [12]. However, few studies have extended this broader view to examine how different patterns of engagement are associated with students’ self-perceived ability to locate, evaluate, and apply online health information. Distinguishing engagement patterns from exposure time is important because self-report measures of eHealth literacy reflect students’ perceived confidence and competence in digital health information practices rather than objectively tested literacy skills. Accordingly, the present study addresses this gap by pairing the Social Networking Usage Questionnaire (SNUQ), which captures multidimensional engagement, with the revised 12-item eHealth Literacy Scale (eHEALS-R), while distinguishing patterns of engagement from the amount of daily social media use.

This study was guided by Pender’s Health Promotion Model (HPM) [13], which describes how cognitive and behavioral factors influence health-related behaviors and information-seeking practices. Within the context of the present study, social networking engagement may represent one of the ways nursing students access, interpret, and interact with online health information. The model is relevant to this study because engagement with digital platforms may be related to students’ perceived ability to evaluate and apply health-related information, in addition to the development of eHealth literacy skills. Previous literature has also suggested that active and meaningful digital usage may support learning experiences, health information evaluation, and health-related behaviors among nursing students [3,8]. Accordingly, HPM provides a useful framework for understanding the relationship between social networking engagement and perceived eHealth literacy among nursing students.

The primary objectives of this study were

- To assess the levels of social networking engagement and perceived eHealth literacy among undergraduate nursing students;
- To examine the relationship between social networking engagement and perceived eHealth literacy;
- To determine whether social networking engagement is independently associated with perceived eHealth literacy after accounting for demographic characteristics and daily social media use.

## 2. Materials and Methods

### 2.1. Study Design

A descriptive cross-sectional correlational design was used to examine the relationship between multidimensional social networking engagement and perceived eHealth literacy among undergraduate nursing students. The study was reported in accordance with the Strengthening the Reporting of Observational Studies in Epidemiology (STROBE) guidelines for cross-sectional studies.

### 2.2. Setting and Participants

A convenience sampling approach was used to recruit undergraduate nursing students from the College of Nursing at a governmental university in Saudi Arabia. A total of 187 undergraduate nursing students were invited to participate through the official academic communication group used by the undergraduate nursing program for routine academic announcements. This program-administered group served as the accessible recruitment frame at the time of survey distribution. The electronic survey link was shared through the group, and QR codes linking to the questionnaire were displayed at the end of selected class sessions to facilitate access. Students were not required to complete the questionnaire during class time, and participation was not linked to any course requirement, assessment, or teaching evaluation. Two reminders were provided during the data collection period. Participation was voluntary and anonymous, and no academic or financial incentives were offered. Of the 187 students invited through this accessible recruitment frame, 146 completed the questionnaire, yielding a response rate of 78%. All 146 completed questionnaires were included in the final analysis. The survey platform did not enforce account-based duplicate control in order to preserve anonymous participation; however, distribution through a single closed program-administered group reduced the likelihood of duplicate submissions.

Eligible participants were students enrolled in the undergraduate nursing program during the study period. In this program, academic levels refer to semester-based progression across the curriculum. Levels 1–2 represent early foundational coursework, Levels 3–5 represent intermediate nursing coursework, and Levels 6–8 represent advanced nursing coursework with greater clinical and professional exposure. The internship level refers to the clinical training period completed after the academic coursework. Data were collected over a two-month period from January to February 2026 using an electronic survey link. Bridging and postgraduate nursing students were excluded to support sample homogeneity.

### 2.3. Measuring Instrument

Data were collected using a structured questionnaire consisting of three sections. The first section collected demographic characteristics, including age, gender, academic level, and average daily social media use. The second section included the SNUQ, a 19-item instrument used to assess patterns of social networking engagement among nursing students. The SNUQ measures four dimensions of social networking use: academic, socialization, entertainment, and informativeness [14]. Items are rated on a 5-point scale ranging from 1 (Never) to 5 (Always), with higher scores indicating greater social networking engagement. The original development study reported good internal consistency, with an overall Cronbach’s alpha of 0.830 [14]. A recent study among nursing students in Saudi Arabia also reported excellent reliability for the SNUQ, with a Cronbach’s alpha of 0.89 [2].

The third section included the eHEALS-R, a revised 12-item scale used to assess students’ perceived eHealth literacy. The scale measures perceived ability to locate, evaluate, and use online health information [15]. Items are rated on a 5-point Likert scale ranging from 1 (Strongly Disagree) to 5 (Strongly Agree), with higher scores indicating greater perceived eHealth literacy.

### 2.4. Ethical Considerations

Ethical approval was obtained from the Research Ethics Committee of Majmaah University, Saudi Arabia (Approval No. MUREC-Dec.14/COM-2025/285; approved on 14 December 2025). Participation was voluntary and anonymous. Electronic informed consent and study information were provided to all participants before completing the survey. Participants were informed about the purpose of the study, confidentiality of the collected data, and their right to withdraw from the study at any time without consequences. Anonymity was maintained throughout the data collection and analysis process. The study was conducted in accordance with the principles of the Declaration of Helsinki.

### 2.5. Data Analysis

Data were analyzed using IBM SPSS Statistics version 26 (IBM Corp., Armonk, NY, USA). Data were screened for completeness before analysis. The final analytic sample comprised 146 participants, all of whom completed the questionnaire in full; therefore, no item-level missing data occurred, and all available cases were included in the analyses. Descriptive statistics, including frequencies, percentages, means, and standard deviations, were used to summarize demographic characteristics and study variables. Academic level was categorized into four groups: Levels 1–2, Levels 3–5, Levels 6–8, and internship level. Because only one participant was in the internship category, this category was combined with Levels 6–8 for regression analysis. Internal consistency of the study instruments was evaluated using Cronbach’s alpha. Pearson’s correlation coefficient was used to examine the relationship between SNUQ and eHEALS-R average scores. Common method bias was assessed using Harman’s single-factor test by entering all scale items into a single-factor solution.

Simple and multiple linear regression analyses were conducted using the eHEALS-R average score, reflecting perceived eHealth literacy, as the dependent variable. The multiple regression model included SNUQ average score, age, gender, academic level, and daily social media use. A post hoc sensitivity analysis was conducted for the adjusted regression model using G*Power version 3.1.9.7 (Heinrich Heine University Düsseldorf, Düsseldorf, Germany). With *n* = 146, α = 0.05, 80% power, one tested variable, and 10 total variables in the model, the minimum detectable individual effect size was f^2^ = 0.05. Accordingly, non-significant associations for demographic variables were interpreted cautiously as an absence of evidence rather than evidence of absence. Regression assumptions were assessed before model interpretation. Variance inflation factors were used to assess multicollinearity, while Q-Q plots, residuals-versus-fitted plots, scale-location plots, residuals-versus-leverage plots, and Cook’s distance were examined to evaluate residual normality, homoscedasticity, and influential observations. Statistical significance was established at *p* < 0.05.

## 3. Results

### 3.1. Participant Characteristics

The study included 146 undergraduate nursing students. Most participants were aged 18–20 years (*n* = 71, 49%), followed by those aged 21–23 years (*n* = 52, 36%). The remaining participants were aged 24 years or older (*n* = 23, 16%). Female students represented 51% of the sample. Participants were distributed across Levels 1–2 (29%), Levels 3–5 (36%), Levels 6–8 (34%), and internship level (1%). Daily social media use was generally high, with 45% of participants reporting 5 or more hours of use per day, and 38% reporting 3–4 h daily (Table 1).

### 3.2. Descriptive Statistics and Reliability of Study Instruments

At the scale level, the SNUQ demonstrated strong internal consistency (Cronbach’s α = 0.902), with a mean score of 3.57 ± 0.79. The eHEALS-R also demonstrated good internal consistency (Cronbach’s α = 0.894), with a mean score of 3.62 ± 0.87 (Table 2). Overall, participants reported moderate-to-high social networking engagement and favorable perceived eHealth literacy. Descriptive item-level statistics are presented in Appendix A Table A1. Among the eHEALS-R items, the highest-rated statement was “I can identify fake or misleading health information online” (M = 3.82), whereas the lowest-rated statement was “I trust information about health I find online” (M = 3.22). Harman’s single-factor test showed that the single-factor solution accounted for 36.0% of the total variance, below the 50% threshold for common method bias.

### 3.3. Correlation Between SNUQ and Perceived eHealth Literacy

**Figure 1:**
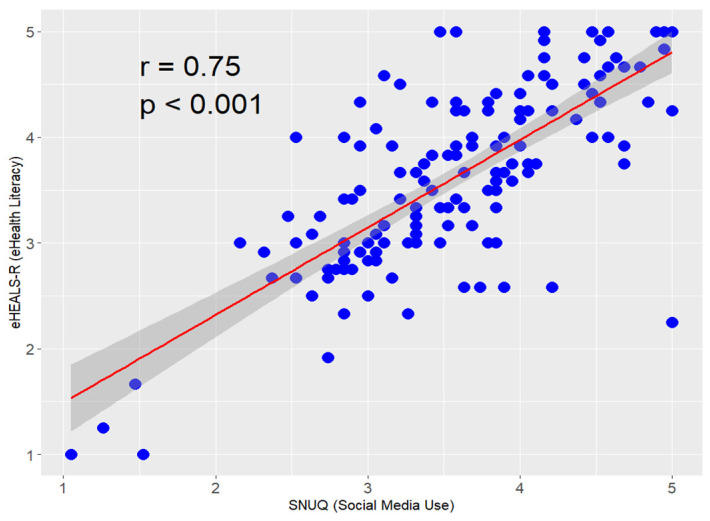
Scatterplot showing the correlation between SNUQ and eHEALS-R scores. Blue dots represent individual observations, the red line represents the fitted linear regression line, and the gray shaded area represents the 95% confidence interval.

Pearson’s correlation analysis showed a strong positive association between SNUQ and eHEALS-R average scores (r = 0.75, *p* < 0.001) (Figure 1). Higher social networking engagement was associated with higher perceived eHealth literacy among participants. At the dimension level, all four SNUQ subscales were positively and significantly correlated with perceived eHealth literacy (all *p* < 0.001). The strongest association was observed for academic use (r = 0.71), followed by entertainment (r = 0.66), socialization (r = 0.58), and informativeness (r = 0.54). Inter-correlations among the four SNUQ dimensions ranged from moderate to moderately strong (r = 0.44–0.68), indicating that the dimensions were related but empirically distinct and did not suggest substantial conceptual redundancy (Table 3).

### 3.4. Regression Analysis of Factors Associated with Perceived eHealth Literacy

In the univariable linear regression analysis, the SNUQ average score was significantly associated with the eHEALS-R average score (Estimate = 0.83; 95% CI: 0.71–0.95; *p* < 0.001), explaining 56% of the variance in perceived eHealth literacy (R^2^ = 0.563). In the multivariable model including age, gender, academic level, and daily social media use, the SNUQ average score remained significantly associated with the eHEALS-R average score (Estimate = 0.83; 95% CI: 0.71–0.96; *p* < 0.001; β = 0.76). The full model explained 58% of the variance in perceived eHealth literacy (R^2^ = 0.58; adjusted R^2^ = 0.55). Demographic variables and daily social media use did not show statistically significant associations with eHEALS-R average scores in the multivariable model (all *p* > 0.05); these non-significant findings were interpreted cautiously in light of the sensitivity analysis (Table 4).

**Figure 2:**
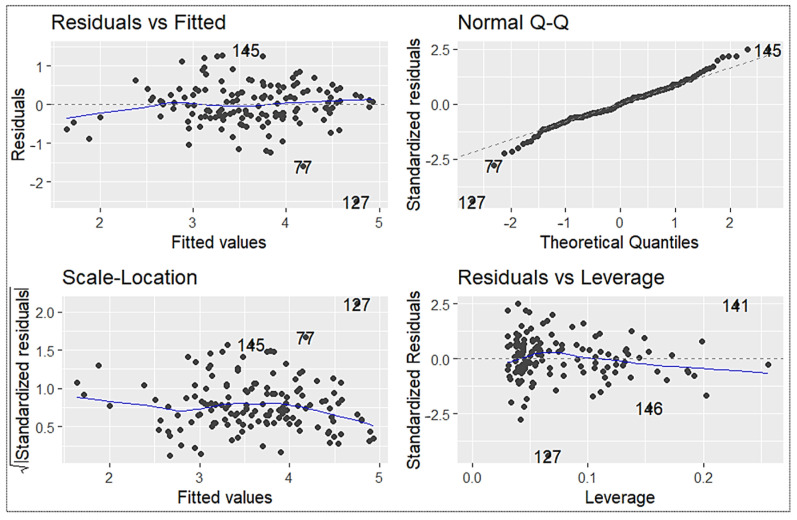
Regression diagnostic plots for the multiple linear regression model. Dots represent individual observations, blue lines show smoothed trends, and dotted reference lines indicate zero residuals or the theoretical normal reference in the Q–Q plot.

Model diagnostics supported the adequacy of the multiple linear regression model. Variance inflation factors ranged from 1.04 to 1.25, indicating no evidence of multicollinearity. Visual inspection of the residuals-versus-fitted plot showed no clear systematic pattern, supporting the assumptions of linearity and homoscedasticity. The normal Q-Q plot showed that residuals were reasonably aligned with the theoretical normal distribution, with only minor deviations at the tails. The scale-location plot did not indicate substantial heteroscedasticity. The residuals-versus-leverage plot showed a small number of observations with relatively higher leverage; however, none appeared to exert undue influence on the regression estimates based on Cook’s distance (Figure 2).

## 4. Discussion

The present study identified a strong positive association between social networking engagement and perceived eHealth literacy among undergraduate nursing students. Students who reported higher engagement with social networking platforms also reported greater confidence in accessing, evaluating, and using online health information. This finding highlights social networking engagement as a meaningful aspect of nursing students’ digital learning and everyday information practices.

This pattern is consistent with studies linking specific digital competencies to eHealth literacy. Sun et al. reported that social media self-efficacy was associated with eHealth literacy among Chinese nursing undergraduates [16]. Jeon and Kim also found that digital literacy was related to eHealth literacy among nursing students in South Korea [17]. These findings indicate that perceived eHealth literacy is closely connected to students’ confidence and capability in using digital environments.

The distinction between exposure time and engagement patterns was evident in the present findings. Social networking engagement remained associated with perceived eHealth literacy in the adjusted model, while daily social media use did not. A similar separation appears in Ibrahim et al., who found that academic and socialization motives for social media use were related to academic performance among nursing students, with the time spent on social media showing limited relevance [2]. Neither the present analysis nor that of Ibrahim et al. identified the duration of use as a meaningful correlate of these outcomes.

The dimension-level findings help clarify the distinction between engagement patterns and exposure time. Although all four SNUQ dimensions were positively associated with perceived eHealth literacy, academic use showed the strongest association. This pattern is meaningful in nursing education because academically oriented social media use may involve course-related discussion, exam preparation, peer learning, and access to health-related educational resources [1,2]. These activities are closely aligned with students’ perceived ability to locate, evaluate, and apply online health information. Rather than suggesting that one form of digital engagement is exclusively important, this finding reinforces the value of distinguishing purposeful academic engagement from general exposure to social media.

However, evidence on social media use and digital competencies is not uniform. Erdat et al. found that social media use was negatively associated with digital literacy among nursing students in Turkey [18]. In the same study, internet self-efficacy was positively associated with digital literacy. This difference may partly stem from how social media use and literacy outcomes were measured: a general use measure can capture passive or unstructured activity, whereas the present study used the SNUQ to assess multidimensional engagement, including academic, informational, social, and recreational use, in relation to perceived eHealth literacy.

The interpretation of these findings should also consider the educational and sociocultural context in which the instruments were used. The SNUQ was selected because it captures multidimensional social networking engagement, including academic, social, entertainment, and informational uses, and moves beyond exposure time as a single indicator. Its previous use among nursing students in Saudi Arabia, along with evidence of pilot clarity and strong internal consistency, provides local support for its suitability within an English-medium nursing education setting [2]. In this study, the SNUQ also demonstrated strong internal consistency, reinforcing its appropriateness for describing patterns of social networking engagement in this undergraduate context. The eHEALS-R likewise offered a structured way to examine students’ perceived eHealth literacy in relation to their digital learning and information-seeking practices.

The item-level findings also provide useful insight into how students perceived their eHealth literacy. Participants reported relatively high confidence in recognizing misleading online health information, but lower confidence in trusting health information available on the Internet. This pattern suggests that students’ perceived eHealth literacy may include a cautious approach to online health content, not simply confidence in digital access. For nursing education, the implication is that digital preparation should be neither limited to accessing information nor focused only on technical use. It should also strengthen students’ judgment in evaluating credibility and applying online health information appropriately, particularly given the prevalence of misinformation in online health environments [5].

Demographic variables and daily social media use did not show statistically significant associations with perceived eHealth literacy in the adjusted model. This result should be interpreted within the context of the single-institution sample and the limited ability to detect smaller individual effects. A similar pattern was reported by Ibrahim et al., whose regression analysis showed no statistically significant associations between selected demographic variables and social media use among nursing students [2]. Other studies, however, have reported different trends. Zerilli et al. observed that eHealth literacy varied by academic level, with more senior nursing students demonstrating greater competence in using, evaluating, and applying online health information [19]. Liu et al. also reported that digital health literacy was associated with individual and behavioral characteristics, including academic performance and health-related lifestyle, while gender was not significant in the multivariable model [8]. These variations suggest that demographic associations with perceived eHealth literacy may vary according to educational context, measurement approach, and students’ patterns of digital learning exposure.

These findings can also be interpreted in light of Pender’s Health Promotion Model, which emphasizes cognitive and behavioral processes that shape health-related actions [13]. In this context, social networking engagement may serve as one behavioral setting in which students encounter, evaluate, and use online health information. This perspective positions perceived eHealth literacy as part of students’ broader confidence in navigating and applying health information within everyday digital environments.

From an educational perspective, the findings suggest that nursing programs may benefit from guiding students toward more purposeful and critical use of digital platforms. Learning activities that involve appraising online health information, identifying misinformation, and discussing digital credibility may help students apply these skills in academic and clinical settings. This approach allows social platforms to be used as spaces for guided learning and peer exchange, while reducing uncritical reliance on unverified content.

Several limitations should be considered. The cross-sectional design does not allow conclusions about the direction of the relationship between social networking engagement and perceived eHealth literacy. Participants were recruited by convenience from a single institution, and the sample was relatively small, which limits generalizability. Both constructs were self-reported, so the eHEALS-R reflects perceived rather than objectively measured competence. This may introduce response bias, including social desirability or overestimation of digital health abilities. Although Harman’s single-factor test was below the 50% threshold, common-method variance remains a consideration because both measures were collected within a single survey.

Future research using longitudinal or interventional designs could help clarify the direction of the relationship between social networking engagement and perceived eHealth literacy. Such studies could also examine whether structured digital learning activities support the development of students’ confidence and competence in evaluating online health information over time. Studies across multiple institutions, with the inclusion of objective or performance-based measures alongside self-report, would further strengthen the evidence.

## 5. Conclusions

A strong positive association was observed between social networking engagement and perceived eHealth literacy among undergraduate nursing students. This association remained significant after accounting for demographic characteristics and daily social media use, suggesting that how students engage with digital platforms may be more relevant to their perceived eHealth literacy than the amount of time they spend online. The stronger association observed for academic use further highlights the value of purposeful digital engagement in nursing education. Integrating eHealth literacy development within digital learning activities may help prepare students to navigate contemporary digital healthcare environments.

## Figures and Tables

**Table 1:** Demographic Characteristics of Participants.

Variables	N (%)
	(N = 146)
Age	
18–20 years	71 (49%)
21–23 years	52 (36%)
24–26 years	12 (8%)
27 years or above	11 (8%)
Gender	
Female	75 (51%)
Male	71 (49%)
Academic Level	
Level 1 to Level 2	42 (29%)
Level 3 to Level 5	53 (36%)
Level 6 to Level 8	50 (34%)
Internship	1 (1%)
Daily Social Media Use (hours/day)	
<1 h per day	7 (5%)
1–2 h per day	18 (12%)
3–4 h per day	56 (38%)
5 or more hours per day	65 (45%)

**Table 2:** Item and Scale-Level Descriptive Statistics & Reliability of Scales.

Variables	Mean (SD)	Cronbach’s Alpha	Number of Items
SNUQ Average Scores	3.57 (0.79)	0.902	19
eHEALS-R Average Scores	3.62 (0.87)	0.894	12

**Table 3:** Pearson Correlations Among SNUQ Dimensions and Perceived eHealth Literacy.

Variables	1	2	3	4	5
1. Academic use	—				
2. Socialization	0.63 **	—			
3. Entertainment	0.68 **	0.66 **	—		
4. Informativeness	0.63 **	0.54 **	0.44 **	—	
5. Perceived eHealth literacy	0.71 **	0.58 **	0.66 **	0.54 **	—

Note: Values are Pearson correlation coefficients. Inter-dimension correlations ranged from 0.44 to 0.68. ** *p* < 0.001.

**Table 4:** Regression Analysis Results for Factors Associated with Perceived eHealth Literacy Based on SNUQ.

	Univariate Linear Regression Model				Multiple Linear Regression Model			
Variables	Estimates	Std. Beta	95% CI	*p*	Estimates	Std. Beta	95% CI	*p*
(Intercept)	0.66	0.00	0.22–1.10	**0.003**	0.70	0.06	0.05–1.34	**0.034**
SNUQ	0.83	0.75	0.71–0.95	**<0.001**	0.83	0.76	0.71–0.96	**<0.001**
Age								
18–20 years					—	—	—	—
21–23 years					0.08	0.09	−0.23–0.39	0.61
24–26 years					−0.30	−0.34	−0.71–0.12	0.16
27 years or above					0.02	0.02	−0.39–0.43	0.99
Gender								
Female					—	—	—	—
Male					0.006	0.007	−0.21–0.23	0.96
Academic Level								
Level 1 to Level 2					—	—	—	—
Level 3 to Level 5					0.001	0.001	−0.25–0.25	0.99
Level 6 to Level 8					0.01	0.01	−0.32–0.35	0.95
Daily Social Media Use (hours per/day)								
<1 h per day					—	—	—	—
1–2 h per day					−0.06	−0.07	−0.58–0.46	0.809
3–4 h per day					−0.13	−0.14	−0.61–0.36	0.609
5+ h per day					−0.03	−0.03	−0.51–0.45	0.91
Observations	146				146			
R^2^/R^2^ adjusted	0.563/0.560				0.581/0.550			

Note: CI = confidence interval; Std. Beta = standardized coefficient. Reference categories were as follows: Age = 18–20 years; Gender = female; Academic level = Levels 1–2; Daily social media use ≤ 1 h per day. Bold *p*-values indicate statistical significance at *p* < 0.05.

## Data Availability

The data presented in this study are available from the corresponding author upon reasonable request. The data are not publicly available due to privacy and ethical restrictions.

## Public Involvement Statement

No public involvement in any aspect of this research.

## Guidelines and Standards Statement

This manuscript was drafted in accordance with the Strengthening the Reporting of Observational Studies in Epidemiology (STROBE) guidelines for cross-sectional research.

## Abbreviations

The following abbreviations are used in this manuscript:

eHEALS-R	Revised eHealth Literacy Scale
SNUQ	Social Networking Usage Questionnaire
SPSS	Statistical Package for the Social Sciences
HPM	Health Promotion Model
CI	confidence interval
Std. Beta	standardized coefficient

## Appendix A

**Table A1:** Item Level Descriptive Statistics & Reliability of Scales.

Variables	Mean (SD)
SNUQ Average Scores	
I use social networking sites to become more sociable.	3.12 (1.23)
I use social networking sites to keep in touch with my relatives	3.60 (1.24)
I use social networking sites to seek help from my teachers	3.34 (1.25)
I use social networking sites for getting job-related information	3.55 (1.46)
I use social networking sites to share new ideas	3.52 (1.41)
I use social networking sites to create my social identity	3.14 (1.36)
I prefer using social networking sites to attending social gatherings	3.31 (1.34)
I use social networking sites to get information regarding current social events	3.73 (1.19)
I use social networking sites for online academic group discussion	3.60 (1.23)
I use social networking sites for reading news	3.79 (1.31)
I use social networking sites for sharing pictures.	3.57 (1.36)
I use social networking sites to do research work	3.47 (1.39)
I use social networking sites to learn about my curricular aspect	3.77 (1.23)
I communicate with my friends via social networking sites for exam preparation	3.82 (1.27)
I use social networking sites to get relief from academic stress	3.68 (1.29)
I use social networking sites for watching movies	3.73 (1.35)
I use social networking sites for collaborative learning	3.66 (1.28)
I use social networking sites to solve my academic problem	3.55 (1.38)
I use social networking sites to look at funny sharing	3.79 (1.29)
eHEALS-R Average Scores	
I know how to use the internet to answer my health questions	3.58 (1.34)
I know where to find helpful health resources online	3.70 (1.23)
I know how to find helpful health resources on social media	3.80 (1.17)
I know how to use online tools to help me make health decisions	3.52 (1.42)
I can tell high quality health resources from low quality health resources online	3.68 (1.25)
I feel confident using information from the internet to make health decisions	3.42 (1.31)
I trust information about health I find online	3.22 (1.35)
I can use technology to improve my health	3.62 (1.29)
I can use mobile apps to track or improve health behaviors	3.68 (1.31)
I can evaluate whether health information shared by influencers is reliable	3.71 (1.25)
I can identify fake or misleading health information online	3.82 (1.10)
I feel confident discussing online health information with healthcare professionals	3.66 (1.36)

## References

[1] Giroux C.M., Moreau K.A. (2022). Nursing students’ use of social media in their learning: A case study of a Canadian school of nursing. BMC Nurs..

[2] Ibrahim I.A., Mohamed M.H.M., Alenezi A. (2024). Exploring the linkages between social media use, self-esteem, and academic performance among nursing students in Saudi Arabia: A descriptive correlational study. Belitung Nurs. J..

[3] Cathala X., Ocho O.N., McIntosh N., Watts P.N., Moorley C. (2023). An exploration of social participation in Caribbean student nurses’ use of social media in their learning journey. J. Adv. Nurs..

[4] O’Connor S., Odewusi T., Smith P.M., Booth R.G. (2022). Digital professionalism on social media: The opinions of undergraduate nursing students. Nurse Educ. Today.

[5] Zafra-Agea J.A., Calvillo-Nuñez N., Gil-Jiménez Ò., Hellín-Pijuan I. (2023). Perception of internet use in relation to health decision-making among nursing students. Eur. J. Investig. Health Psychol. Educ..

[6] Ye T., Luo J., Chen Y., Huang Y., He M., Yang J., Wang T., Yao Q., Qu Y., Yang Z. (2025). The influence of meaning in life on smartphone addiction among nursing undergraduates: The mediating roles of professional identity and achievement motivation. BMC Nurs..

[7] Zhou Z., Liu H., Zhang D., Wei H., Zhang M., Huang A. (2022). Mediating effects of academic self-efficacy and smartphone addiction on the relationship between professional attitude and academic burnout in nursing students: A cross-sectional study. Nurse Educ. Today.

[8] Liu L., Fu M., Wu J., Wang H., Zhao J., Chen P., Cao J., Zhang W., Lin Q., Li L. (2024). Digital health literacy among undergraduate nursing students in China: Associations with health lifestyles and psychological resilience. BMC Med. Educ..

[9] Sinan O., Ayaz-Alkaya S., Akca A. (2023). Predictors of eHealth literacy levels among nursing students: A descriptive and correlational study. Nurse Educ. Pract..

[10] Atallah S., Mansour H., Dimassi H., Kabbara W.K. (2023). Impact of social media education on antimicrobial stewardship awareness among pharmacy, medical and nursing students and residents. BMC Med. Educ..

[11] Panczyk M., Cieślak I., Kirwan M., Wawrzuta D., Małkowski P., Dobrowolska B., Dyk D., Gaworska-Krzemińska A., Grochans E., Kózka M. (2025). Assessment of social media literacy among nursing students in Poland: Psychometric evaluation of the Polish version of the Perceived Social Media Literacy Scale. Teach. Learn. Nurs..

[12] Gum L., Brown A., Royals R., Matricciani L., Kelly M.A. (2024). Digital professionalism in preregistration nursing and midwifery students: A scoping review to explore perceptions of professionalism when using social media. Nurse Educ. Pract..

[13] Murdaugh C.L., Parsons M.A., Pender N.J. (2019). Health Promotion in Nursing Practice.

[14] Gupta S., Bashir L. (2018). Social Networking Usage Questionnaire: Development and validation in an Indian higher education context. Turk. Online J. Distance Educ..

[15] Norman C., Skinner H., Milanti A., Chan D.N.S., So W.K.W. (2025). eHealth Literacy Scale Manual: eHEALS and eHEALS-R.

[16] Sun H., Qian L., Xue M., Zhou T., Qu J., Zhou J., Qu J., Ji S., Bu Y., Hu Y. (2022). The relationship between eHealth literacy, social media self-efficacy and health communication intention among Chinese nursing undergraduates: A cross-sectional study. Front. Public Health.

[17] Jeon J., Kim S. (2022). The mediating effects of digital literacy and self-efficacy on the relationship between learning attitudes and eHealth literacy in nursing students: A cross-sectional study. Nurse Educ. Today.

[18] Erdat Y., Sezer Ceren R.E., Ozdemir L., Uslu-Sahan F., Bilgin A. (2023). Influence of technical, cognitive and socio-emotional factors on digital literacy in nursing students assessed using structural equation modeling. Nurse Educ. Today.

[19] Zerilli I., Bulfone G., Capizzello D., Gambera A., Fazzino V., Sudano M., Vinci A., Ingravalle F., Maurici M. (2026). Understanding health literacy and eHealth literacy in nursing students: A cross-sectional cluster analysis. Nurs. Rep..

